# Adherence to Chronic Hepatitis C Treatment Regimen: First Report From a Referral Center in Iran

**DOI:** 10.5812/hepatmon.11038

**Published:** 2013-06-23

**Authors:** Saeedeh Ravi, Mohsen Nasiri Toosi, Iman Karimzadeh, Mehdi Ahadi-Barzoki, Hossein Khalili

**Affiliations:** 1 Department of Clinical Pharmacy, Faculty of Pharmacy, Tehran University of Medical Sciences, Tehran, IR Iran; 2 Department of Clinical Pharmacy, Pharmaceutical Sciences Branch, Islamic Azad University, Tehran, IR Iran; 3 Department of Gastroenterology, Faculty of Medicine, Tehran University of Medical Sciences, Tehran, IR Iran; 4 Department of Clinical Pharmacy, Faculty of Pharmacy, Zanjan University of Medical Sciences, Zanjan, IR Iran

**Keywords:** Hepatitis C, Chronic, Medication Adherence, Iran

## Abstract

**Background:**

Various aspects of adherence to HCV treatment such as frequency, risk factors as well as causes of non-adherence, and its real role in clinical and virological outcome of the infected patients have remained largely unknown.

**Objectives:**

The current study aimed to evaluate patients’ adherence to anti-HCV medications in Iran.

**Materials and Methods:**

From October 2010 to March 2011, socio-demographic characteristics, features of HCV infection, clinical properties, and habitual history of 190 patients were collected. Adherence of each patient to anti-HCV medications was determined at months 1, 3, and 6 of treatment by self-reporting and pill or empty ampoule counting. Adherence to anti-HCV treatment regimen was determined based on the 80/80/80 rule.

**Results:**

Adherence rate to interferon, ribavirin, or a combination of them over the first 6 months of therapy in Iranian HCV patients measured by both methods of self-reporting and pill counting were 35.4-65.8%, 46.3-56.8%, and 28.4-51.1%, respectively. Delay in receiving new prescription, financial issues, and adverse drug reactions were the most common causes of non-adherence in the patients. Adherence to ribavirin was identified as an independent predictor of achieving the end of treatment response, or sustained virological response.

**Conclusions:**

The rate of adherence to interferon and ribavirin varied significantly according to the method of calculation. Over the treatment course, adherence to interferon alpha and ribavirin, each or their combination, diminished significantly.

## 1. Background

In developing countries, chronic hepatitis C has been considered as the major cause of cirrhosis and hepatocellular carcinoma which need liver transplantation (1, 2). Based on World Health Organization (WHO) reports, the global prevalence of hepatitis C virus (HCV) infection is 2.2% to 3% (3, 4). An overall HCV seroprevalence in the general population of Iran has been reported to be 0.5%. Approximately 250,000 of Iranians are currently living with chronic hepatitis C (5). By the standard treatment regimen, 40-54% of patients with HCV genotype 1 and 65-82% of those infected with HCV genotypes 2 or 3 achieve sustained virological response (SVR) (6). Response to anti-HCV treatment has been attributed to both host characteristics and viral factors (7). Patient adherence to treatment regimen plays a pivotal role in the management of chronic HCV infection (8-11). According to the National Institutes of Health Consensus Statement on the Management of hepatitis C in 2002, patient adherence to prescribed treatment regimen plays a pivotal role in the management of chronic HCV infection (2). A review of related literature published in 2002 to 2007 confirmed this issue (1). McHutchison et al. in 2002 demonstrated that patients who received ≥80% of the prescribed doses of pegylated interferon and ribavirin for ≥80% of the scheduled therapy duration had higher SVR rates than less adherent patients (63% versus 52%, respectively) (4). The numerous hematological, physical, and neuropsychiatric side effects of anti-HCV medications which occur in nearly all patients can potentially lead to dose reductions as well as early treatment discontinuation. In clinical trials 15-20%, and in clinical practice more than 25% of patients have been reported to discontinue their anti-HCV treatment (3). Various aspects of adherence to HCV treatment such as frequency, risk factors as well as causes of non-adherence, and its real role in clinical and virological outcome of the infected patients have remained largely unknown.

## 2. Objectives

The current study aimed to determine the profile of patients’ adherence to anti-HCV medications by two different methods and assess risk factors for non-adherence to anti-HCV medications in a referral center in Iran. To the best of our knowledge, this is the first comprehensive report from this area.

## 3. Materials and Methods

This prospective, observational study was performed during a 1.5 year period from October 2010 to March 2011 in the Hepatitis Clinic of Imam Khomeini Hospital, main and referral teaching hospital affiliated to Tehran University of Medical Sciences, Tehran, Iran. The Institutional Review Board (IRB) and the Medical Ethics Committee of the hospital approved the study. All patients gave their written consent form. All adult (≥ 18 years) HCV infected patients candidate to receive anti-HCV treatment regimen including conventional or pegylated interferon alpha plus ribavirin were recruited. During the study period, 190 patients with inclusion criteria were enrolled. Treatment protocol was according to the last version of the American Association for the Study of Liver Diseases (AASLD) practice guideline (12). The patients’ data including socio-demographic characteristics (age, sex, educational status, occupation, marital status, living condition, history of prison), features of HCV infection (genotype, subtype, fibrosis stage and viral load at diagnosis, source of infection, virological response), clinical properties (previous anti-HCV treatment course, current anti-HCV treatment regimen, concomitant medications, adverse reactions to anti-HCV medications, any dose reduction or discontinuation of (peg) interferon alpha and/or ribavirin due to adverse reactions according to standard algorithms or manufacturer recommendations (13-15), co-morbidities), and habitual history (alcohol consumption and/or smoking) were collected. Adherence of each patient to anti-HCV medications were determined at months one, three, and six of treatment by self-reporting and pill or empty ampoule counting. In self-reporting strategy, the following questions were asked from each patient: frequency and date of (peg) interferon alpha or its pegylated products injection during the past forth weeks, number and date of ribavirin capsules taken in the morning and evening during the past seven days, frequency, reason for taking (peg) interferon alpha injections and/or ribavirin capsules more/less than prescribed, family awareness about status of patient disease and/or treatment, dependency on others in taking their anti-HCV medications, and using auxiliary means to remember anti-HCV medications (e.g. timed pill boxes, calendar). In addition to self-reporting, adherence to (peg) interferon alpha and ribavirin was also assessed by empty container and pill counting, respectively. The number of anti-HCV medications taken during the past forth weeks was determined by counting the number of ribavirin capsules or empty (peg) interferon alpha containers (ampoule, vial, or syringe) returned after forth weeks on the interview day. Adherence to anti-HCV treatment regimen was determined by the 80/80/80 rule. It was defined as receiving at least 80% of the prescribed dose of (peg) interferon alpha along with at least 80% of the prescribed dose of ribavirin for at least 80% of the planned treatment course (10). Criteria of virological response were defined according to the last version of the AASLD practice guideline (12).

### 3.1. Statistical Analysis

Categorical data were reported as a percentage and continuous variables were expressed as means ± standard deviations (SD). The rate of adherence to anti-HCV medications, the difference between mean adherence rate to combination anti-HCV treatment regimen during the first six months measured by pill counting and patient self-reporting, possible association of adherence, and virological response were assessed by Chi-square test. Multiple variate logistic regression analysis was used to compare different socio-demographic, clinical, and financial characteristics of adherent and non-adherent patients to anti-HCV treatment regimen during the first 6 months of treatment. Odds ratios (OR) and their 95% confidence intervals (CI) were calculated for each variable. It was also used to assess probable factors contributing to SVR or end of treatment response (ETR). Values of variance inflation factor (VIF) over 10 and simultaneously, tolerance levels less than 0.1 were regarded as the presence of multicollinearity. SPSS software version 15 was employed to perform all statistical analyses. P-values less than 0.05 were considered as statistically significant.

## 4. Results

Among 190 study population, about ninety percent (91.1%) were male. Injection of illicit drugs (76.3%) was the most frequent source of HCV infection. No patient was co-infected with hepatitis B virus. Twenty nine patients had a history of anti-HCV treatment including conventional interferon alpha plus ribavirin (24 cases, 82.8%) and pegylated interferon alpha-2a plus ribavirin (5 cases, 17.2%) before recruitment. Anti-HCV regimen adverse reactions (e.g. hematologic dyscrasias, psychiatric disorders) were considered as the reason for dose reduction of (peg) interferon alpha and ribavirin in 3 and 10 patients, respectively. (Peg) interferon alpha and ribavirin were discontinued early or temporally due to adverse reactions in 17 and 15 subjects, respectively. Of 190 patients, family of 159 (83.7%) were aware of their diseases or treatment. Demographic, social, and clinical characteristics of the study population are shown in Table 1.

**Table 1.** Demographic, Social, and Clinical Characteristics of the Study Population (n = 190)

Parameter	No. (%)
**Gender**	
Male	173 (91.1)
Female	17 (8.9)
**Age, y**	
Mean ± SD	39.4 ± 10.87
Range	22-81
**Educational status**	
Illiterate	18 (9.5)
School grade	164 (86.3)
Academic grade	8 (4.2)
**Occupation**	
Employed	142 (74.7)
Unemployed	48 (25.3)
**Marital status**	
Single	89 (46.8)
Married	101 (53.2)
**Living condition**	
Alone	43 (22.6)
With family	147 (77.4)
**Co-administered medications**	
Mean ± SD	4.51 ± 2.94
Range	2-18
**Route of HCV transmission**	
Injection of illicit drugs	145 (76.3)
Tattooing	14 (7.4)
Others	12 (6.3)
Unknown	19 (10)
**HCV genotype**	
1	81 (42.6)
2	2 (1.1)
3	104 (54.7)
Unknown	4 (2.1)
**Subtype**	
a	155 (81.6)
b	6 (3.2)
Unknown	29 (15.3)
**Pre-treatment viral load (IU/ml)**	
≤ 800,000	104 (54.7)
> 800,000	73 (38.4)
Unknown	13 (6.8)
**Smoking**	
Never	28 (14.7)
Previous	17 (8.9)
Current	145 (76.3)
**Alcohol consumption**	
Never	67 (35.3)
Previous	116 (61.1)
Current	7 (3.7)
**History of prison**	113 (59.5)
**History of anti-HCV treatment (relapse/non-responder)**	29 (15.3)
**Comorbidities**	126 (66.32)

Pegylated interferon alpha-2a plus ribavirin was the most frequent current anti-HCV treatment regimen (69.5%), followed by conventional interferon alpha plus ribavirin (20%) and pegylated interferon alpha-2b plus ribavirin (10.5%). One hundred and thirty two (69.5%) of the patients took anti-HCV medications by themselves and the remaining (30.5%), received assistance for their prescribed medications. Timed pill boxes, calendar, or reminding by another person were assistance methods in 62 (32.6%) of the patients. Regarding the last documented virological responses of the patients to anti-HCV treatment regimen during the study period, sixty one (40.9%) and 13 (8.7%) patients achieved ETR and SVR, respectively. Six (4%) and one (0.7%) patients were identified as non-responders and relapsers, respectively. Table 2 summarizes and compares the rate of adherence to anti-HCV medications according to patient self-reporting as well as pill counting methods at months one, three, and six of treatment. Although the rate of self-reported adherence to treatment decreased from 98.42% in month one to 67.9% in month three and to 32.1% in month six, but these differences were not statistically significance (P = 0.196 and P = 0.23, respectively). In contrast to self-reporting method, the decrease in adherence rate to treatment determined by pill count method both in month 3 (40%) and month 6 (29.5%) compared to their counterparts in the previous month was statistically significant (P = 0.003 and P < 0.001, respectively). Unlike month one, the difference of adherence rates to combination treatment determined by self-reporting and pill counting methods in months three and six were statistically significant (P < 0.001). Furthermore, the mean adherence rate to combination treatment during the first 6 months calculated by pill counting was significantly lower than that of the one determined by self-reporting (28.4% versus 51.1%, respectively; P < 0.001).

**Table 2.** Patient Adherence Rate to (peg) Interferon Alpha, Ribavirin, and Its Combination at Months 1, 3, and 6 of Treatment Determined by Self-reporting and Pill Counting Methods

Anti-HCV medication	Adherence, Self-reported (%)	Adherence, Pill counting (%)	P value
**Month 1**			
(Peg) Interferon alpha	189/190 (99.5)	166/190 (87.4)	0.126
Ribavirin	189/190 (99.5)	178/190 (93.7)	0.063
Combination	187/190 (98.4)	164/190 (86.3)	0.318
**Month 3**			
(Peg) Interferon alpha	144/190 (75.8)	101/190 (53.2)	< 0.001
Ribavirin	144/190 (75.8)	124/190 (65.3)	< 0.001
Combination	129/190 (67.9)	88/190 (46.3)	< 0.001
**Month 6**			
(Peg) Interferon alpha	78/190 (41.1)	42/190 (22.1)	< 0.001
Ribavirin	94/190 (49.5)	73/190 (38.4)	< 0.001
Combination	61/190 (32.1)	32/190 (16.8)	< 0.001

The mean adherence rate of interferon alpha during the first six months determined by self-reporting was significantly higher than that of ribavirin (65% versus 53.8%, P < 0.001). In contrast to self-reporting method, adherence rates to ribavirin at months three and six as well as mean rate during the first six months calculated by pill counting were significantly higher than that of the interferon alpha at the same time points (65.3%, 38.4%, and 46.3% versus 53.2%, 22.1%, and 35.4%, respectively; P < 0.001). Different characteristics of adherent patients with non-adherent patients to anti-HCV treatment regimen determined by pill counting method were compared and summarized in Table 3. No socio-demographic, clinical, and financial parameters of the patients are associated with adherence to anti-HCV treatment regimen. Similar results were observed when adherence rate was calculated by patient self-reporting method (data not shown). There was no multicollinearity between the above independent parameters.

**Table 3.** Demographic, Social, and Clinical Characteristics of Adherent and Non-adherent Patients to Anti-HCV Treatment Regimen (n = 190)

Parameter	Adherent(n = 54)	Non-adherent(n = 136)	OR(95% CI)	P value
**Sex**			0.102 (0.01-1.08)	0.058
Male,No.(%)	51 (94.4)	122 (89.7)		
Female, No. (%)	3 (5.6)	14 (10.3)		
**Age, y, Mean ± SD**	38.87 ± 10.02	39.61 ± 11.2	1.005 (0.965-1.046)	0.818
**Weight, kg, Mean ± SD**	75.46 ± 13.83	74.87 ± 14.91	1.009 (0.982-1.036)	0.53
**Educational status**			0.491 (0.162-1.486)	0.208
Illiterate, No. (%)	6 (11.1)	12 (8.8)		
School grade, No. (%)	47 (87)	117 (86)		
Academic grade, No. (%)	1 (1.9)	7 (5.1)		
**Occupation**			0.691 (0.234-2.035)	0.502
Employed, No. (%)	41 (75.9)	101 (74.3)		
Unemployed, No. (%)	13 (24.1)	35 (25.7)		
**Marital status**			1.311 (0.526-3.272)	0.561
Single, No. (%)	20 (37)	69 (50.7)		
Married, No. (%)	34 (63)	67 (49.3)		
**Living condition**			1.223 (0.399-3.747)	0.725
Alone, No. (%)	10 (18.5)	33 (24.3)		
With family, No. (%)	44 (81.5)	103 (75.7)		
**Mean monthly income, $**			1.153 (0.548-2.423)	0.708
< 25, No. (%)	5 (9.3)	14 (10.3)		
25-125, No. (%)	38 (70.4)	89 (65.4)		
> 125, No. (%)	11 (20.4)	33 (24.3)		
**History of prison, No. (%)**	35 (64.8)	78 (57.4)	2.069 (0.806-5.313)	0.131
**Alcohol consumption**			0.657 (0.262-1.646)	0.37
None, No. (%)	20 (37)	47 (34.6)		
Current or previous, No. (%)	34 (63)	89 (65.4)		
**Smoking**			0.085 (0.006-1.134)	0.062
None, No. (%)	7 (13)	21 (15.4)		
Current or previous, No. (%)	47 (87)	115 (84.6)		
**HCV genotype**			2.031 (0.957-4.307)	0.065
1, No. (%)	17 (31.5)	65 (47.8)		
Non-1, No. (%)	37 (68.5)	67 (49.3)		
Unknown, No. (%)	0	4 (2.9)		
**Route of HCV transmission**			0.287 (0.078-1.059)	0.061
Injection of illicit drugs, No (%)	46 (85.2)	99 (72.8)		
Non-injection of illicit drugs, No (%)	4 (7.4)	22 (16.2)		
Unknown (%)	4 (7.4)	15 (11)		
**Anti-HCV treatment regimen**			0.562 (0.216-1.46)	0.237
Conventional interferon alpha plus Ribavirin, No. (%)	13 (24.1)	25 (18.4)		
Pegylated interferon alpha plus Ribavirin, No. (%)	41 (75.9)	111 (81.6)		
**Previous anti-HCV treatment course (Mean ± SD)**	4 (7.4)	25 (18.4)	0.399 (0.117-1.359)	0.142
**Concomitant diseases (Mean ± SD)**	0.96 ± 1.027	1.26 ± 1.186	0.756 (0.512-1.118)	0.162
**Co-administered medications (Mean ± SD)**	4.22 ± 3.142	4.63 ± 2.862	0.887 (0.741-1.062)	0.191
**Adverse drug reactions (Mean ± SD)**	15.41 ± 9.663	15.43 ± 10.228	1.038(0.99-1.088)	0.12
**History of psychiatric disease, No. (%)**	6 (11.1)	23 (16.9)	0.735(0.216-2.495)	0.621
**Family awareness about patient disease or treatment, No. (%)**	48 (88.9)	111 (81.6)	1.089(0.325-3.647)	0.891
**Assistance of others in taking anti-HCV medications, No. (%)**	21 (38.9)	37 (27.2)	2.69(0.636-11.366)	0.178
**Using auxiliary methods to remember anti-HCV medications, No. (%)**	22 (40.7)	40 (29.4)	1.012(0.23-4.349)	0.76

Causes of non-adherence to anti-HCV medications reported by patients are shown in Table 4. Delay in receiving new prescription was reported as the most common cause of non-adherence to (peg) interferon alpha (31.62%) and ribavirin (20%).

**Table 4.** Causes of Non-Adherence to Anti-HCV Medications Reported by Patients

(Peg) Interferon alpha	No. (%)
Delay in receiving new prescription	43 (31.6)
Financial issues	25 (18.4)
Adverse drug reaction	20 (14.7)
Unavailability of drug	9 (6.6)
Travelling	9 (6.6)
Drug loss (spill) during preparation for injection	7 (5.2)
Fed up with using drugs	7 (5.2)
Feeling ill	6 (4.4)
Forgetfulness	4 (2.9)
Intentional	2 (1.5)
Others ^a^	4 (2.9)
**Ribavirin**	
Delay in receiving new prescription	25 (20)
Adverse drug reaction	24 (19.2)
Financial issues	23 (18.4)
Forgetfulness	18 (14.4)
Travelling	11 (8.8)
Feeling ill	8 (6.4)
Unavailability of drug	4 (3.2)
Fed up with using drugs	4 (3.2)
Intentional	3 (2.4)
Others ^b^	5 (4)

^a^ Including participation in Narcotics Anonymous (NA) meetings (n = 2), and being imprisoned temporary (n = 2)

^b^Including participation in NA meetings (n = 2), being imprisoned temporary (n = 2), and missing capsules (n = 1)

Patients who had taken ≥80% doses of (peg) interferon alpha, achieved significantly higher ETR or SVR rate than those given <60% and 60-79% doses of (peg) interferon alpha (57.3% versus 22.7% [P < 0.001] and 57.3% versus 20% [P = 0.026], respectively). Similar patterns were identified for ribavirin. In this regards for example, ETR or SVR rate in patients who took ≥80% dose of ribavirin was significantly higher than in the ones who received < 60% and 60-79% dose of ribavirin (66.7% versus 13.3% and 66.7% versus 20%, respectively; P = 0.001 for both). In contrast, no statistically significant difference in the rate of ETR or SVR was observed between patients given <60% and 60-79% dose of (peg) interferon alpha or ribavirin. Virological response was significantly more in adherent than non-adherent patients compared to (peg) interferon alpha (57.3% versus 42.7%), ribavirin (66.7% versus 33.3%), and its combination (72% versus 28%) during the first 6 months achieved ETR or SVR (P < 0.001 for all comparisons). However, after adjustment for gender, age, weight, HCV genotype, viral load, serum alanine aminotransferase level, and liver fibrosis stage at pretreatment, adherence to (peg) interferon alpha, ribavirin or combination of them, only adherence to ribavirin during the first six months (OR = 3.295, 95% CI = 1.184-9.168, P = 0.022) was significantly associated with achieving ETR or SVR. No multicollinearity was identified between studied independent variables (Table 5).

**Table 5.** Demographic, Clinical, and Paraclinical Characteristics of Patients Achieved or Failed to Achieve ETR or SVR During the Study Period

Parameter	Achieved ETR or SVR (n = 75)	Not achieved ETR or SVR (n = 74)	OR (95% CI)	P value
**Sex**			1.207 (0.35 - 4.16)	0.766
Male, No (%)	68 (90.7)	66 (89.2)		
Female, No (%)	7 (9.3)	8 (10.8)		
**Age, y**			0.524 (0.235 - 1.166)	0.113
< 40, No (%)	49 (65.3)	35 (47.3)		
≥ 40, No (%)	26 (34.7)	39 (52.7)		
**Weight, kg**			0.654 (0.294 - 1.453)	0.297
≤ 75, No (%)	47 (62.7)	40 (54.1)		
> 75, No (%)	28 (37.3)	34 (45.9)		
**HCV genotype**			1.888 (0.711 - 5.017)	0.202
1, No (%)	21 (28)	44 (59.5)		
Non-1, No (%)	53 (70.7)	28 (37.8)		
Unknown, No (%)	1 (1.3)	2 (2.7)		
**Pre-treatment viral load, IU/ml**			1.004 (0.539 - 1.869)	0.99
≤ 800,000, No (%)	43 (57.3)	34 (45.9)		
> 800,000, No (%)	27 (36)	34 (45.9)		
Unknown, No (%)	5 (6.7)	6 (8.1)		
**Pre-treatment serum alanine aminotransferase level, IU/l**			0.932 (0.497 - 1.746)	0.825
< 3 times higher than the upper limit of normal, No (%)	47 (62.7)	51 (68.9)		
≥ 3 times higher than the upper limit of normal, No (%)	25 (33.3)	17 (22.9)		
Unknown, No (%)	3 (4)	6 (8.1)		
**Pretreatment liver fibrosis stage**			1.149 (0.62 - 2.129)	0.66
≤ 2, No (%)	11 (14.7)	19 (25.7)		
> 2, No (%)	6 (8)	16 (21.6)		
Unknown, No (%)	58 (77.3)	39 (52.7)		
**(Peg) Interferon alpha adherence **			1.868 (0.556 - 6.275)	0.312
< 80, No (%)	32 (42.7)	53 (71.6)		
≥ 80, No (%)	43 (57.3)	21 (28.4)		
**Ribavirin adherence**			3.295 (1.184 - 9.168)	0.022
< 80, No (%)	25 (33.3)	49 (66.2)		
≥ 80, No (%)	50 (66.7)	25 (33.8)		
**Combined anti-HCV adherence**			0.91 (0.184 - 4.508)	0.908
< 80, No (%)	54 (72)	61 (82.4)		
≥ 80, No (%)	21 (28)	13 (17.6)		

## 5. Discussion

The adherence rate to interferon, ribavirin, or combination of them over the first six months of therapy in Iranian HCV patients measured by both methods of self-reporting and pill counting were 35.4-65.8%, 46.3-56.8%, and 28.4-51.1%, respectively. Adherence to interferon and ribavirin has been reported from 54.1% to 95% in different populations (16-18). This wide variation in rate of adherence to anti-HCV medications alone or in combination could be attributed to several factors such as differences in methods of calculating adherence, duration of follow-up, probable confounders of adherence, and socio-cultural and economical features. The adherence rate to anti-HCV medications alone or in combination determined by a patient self-reporting over the initial 6 months of treatment was significantly higher than the one measured by pill counting in the present study. Studies on HIV antiretroviral have demonstrated that self-reporting overestimates adherence in comparison to electronic monitoring (19-21). Apart from overestimation, reliability of the self-reporting questionnaire closely depends on level of literacy and cognitive ability of individuals. Despite these issues, a meta-analysis of 65 studies has demonstrated that self-reporting is a valid method for assessing adherence to antiretroviral medications (22). In the current study, adherence to both interferon alpha and ribavirin alone or in combination decreased significantly over the treatment course. This decremental pattern in the rate of adherence to anti-HCV medications has been also reported in other surveys (8, 20, 23). Similar pattern was observed for medications of other chronic diseases such as antiretroviral (24), antihypertensive (25), and lipid-lowering agents (25, 26). Comparing the mean adherence rate to ribavirin and interferon alpha within the first 6 months of treatment in the present study showed conflicting results. Previous studies clearly demonstrated that interferon adherence was higher than ribavirin adherence throughout the chronic HCV treatment course (8, 18, 20, 23). They attributed these findings to the more complexity of ribavirin (twice daily oral dosing) compared to peginterferon regimen (once weekly subcutaneous injection) (23). However, it is not exactly the case for the current study population since 20% of individuals received conventional interferon alpha that requires subcutaneous injections 3 times a week. Furthermore, interferon alpha is much more costly than ribavirin monthly. This is confirmed by the fact that financial issues were reported by patients as the second versus third most common cause of non-adherence to interferon alpha and ribavirin, respectively. Another probable explanation for these controversies might be difference in the method of assessing adherence. Most studies used self-reported questionnaire or pharmacy refill data to determine adherence to interferon. In assessing interferon adherence by pill count method in the current study, patients were asked to bring back empty (peg) interferon syringes, vials, or ampoules at each visit. Difficulty in transporting vehicles of (peg) interferon as well as concerning about needle sticking of others especially family members might discourage patients from collaborating with their physician. This might result in underestimation of adherence to interferon measured by the pill count approach. Interestingly, when data obtained from patient self-report questionnaire were considered, no statistically significant difference was observed between adherence rate of (peg) interferon and ribavirin.

Patient`s adherence to planned treatment regimen has demonstrated to be associated with favorable virological responses such as SVR (27). Due to the likelihood of overestimating adherence by self-reporting method, adherence data determined by pill counting were considered for assessing their probable association with SVR or ETR in the current study. We found that adherence to anti-HCV medications alone or in combination significantly associated with higher rates of ETR or SVR. However, after controlling for other variables, only patient adherence to ribavirin was identified as an independent predictor of ETR or SVR. This is in accordance with results of other studies indicating that appropriate consumption of anti-HCV medications particularly ribavirin plays an important role in achieving SVR and preventing relapse (28-31). Various socio-demographic, clinical, and financial parameters of patients have been reported as independent risk factors for non-adherence to anti-HCV medications. Early report from McHutchison et al. in 2002 identified that older patients as well as individuals with advanced stages of liver fibrosis were significantly less adherent to treatment regimen including conventional interferon alpha-2b and ribavirin (10). Interestingly, in another study, only regular illicit drug users had significantly less adherent to anti-HCV treatment regimen (32). As only 11 (5.8%) of the patients in the current study were currently drug abuser, analysis of this item was not statistically feasible. However, the current study also identified no statistically significant association between history of illicit drug use as well as psychiatric disease and non-adherence to anti-HCV medications. In the recently published study, history of psychiatric diseases including bipolar disorder, depression, and schizophrenia or methadone use were not risk factors to non-adherence (23). Although family awareness about patient disease or treatment, assistance of others in taking anti-HCV medications and using auxiliary methods to remember anti-HCV medications were higher in adherent than non-adherent individuals, but these differences were not statistically significant. To our best knowledge, these issues were not considered in other relevant studies. Just, Cacoub et al in 2008 reported that therapeutic education by healthcare professionals other than the prescribing physician maintained adherence to bitherapy and tended to improve SVR after six months in patients with genotype 2/3 HCV infection (8). Patients in the current survey reported delay in receiving new prescription as the main cause of their non-adherence to both prescribed interferon alpha and ribavirin. This delay might be due to several reasons such as concurrency of visits with official or unplanned holidays, occasional change in visit program of the clinic, unavailability of required laboratory tests at visit time, and being too busy. According to the fact that more than three-fourths (76.8%) of the study population had average monthly income less than $200, it is not surprising that financial issues were addressed as one of the three most frequent causes of non-adherence. Interferon loss (spill) during preparation for injection might be indicative of patients` fear or difficulty with subcutaneous self-injections at home. In our recently published study, adverse drug reactions (26.1%), forgetfulness (15.4%), and unavailability to antiretroviral (13%) were reported as the major reasons for non-adherence to highly active antiretroviral therapy (HAART) in Iranian HIV/AIDS patients (33). McHutchison et al. in 2002 suggested that the most common causes of non-adherence to HAART are forgetfulness, being too busy, or feeling ill which appear to be extrapolatable to chronic HCV infection treatment (10). By using measures such as patients` education according to their cultural and educational status, enhancing family and social support, simplifying dosing schedules, offering medication reminder tools, and improving relationship between patient and health-care providers especially physicians and pharmacists, adherence to HCV treatment can be improved.

The present study had several limitations. First, HIV co-infected patients were not evaluated because they were routinely referred to another clinic. In addition, more than 90% of the study population was male. Furthermore, the survey was performed in a single center, and the results may be susceptible to center bias. Thus, regarding probable co-infections, gender, and performing in a single center, results of this survey might not be extrapolatable to a real-world setting of HCV-infected patients even in the population in Iran. It has been shown that HAART could complicate treatment of HCV infection through augmenting ribavirin side effects (e.g. severe anemia) and or inducing liver toxicity which subsequently causes ribavirin or (peg) interferon dose reduction or early discontinuation. Therefore, it is not surprising that virological response to HCV treatment in HIV co-infected patients has been reported to be lower than that of HCV mono-infected individuals [27-40% (34-36) versus 54-56% (37, 38), respectively]. However, the real clinical effects of HAART on adherence to HCV treatment and vice versa have not been elucidated and further investigations in this area are required. Second, the limited follow-up duration did not allow us to determine long-term virological responses to HCV treatment in all patients. Therefore, virological response of 41 (21.58%) individuals were unknown and just 8.7% of patients achieved SVR; while the rate of SVR reported from our population has ranged from 50% to 95.6% (39-42). Only integrating ETR with SVR data enabled us to perform statistical analysis of the probable association of adherence to treatment and virological response. Third, due to the fact that adherence to anti-HCV medications was determined over the initial 6 months of treatment, evaluating the probable effects of late adherence of 42.6% of the patients with genotype 1 HCV (who require a 48-week treatment course) on virological response was not feasible. Finally, the current research was unable to exactly separate rates of missed doses from dose reductions or early treatment discontinuation due to adverse reactions. Therefore, the available data were a combination of persistence (duration on treatment) and adherence (the rate of prescribed doses taken during that time). In contrast, most relevant studies have evaluated exclusively adherence (missed doses) to anti-HCV medications (7).

In conclusion, it was demonstrated that the rate of adherence to (peg) interferon and ribavirin varied significantly according to method of measurement. Over the treatment course, adherence to (peg) interferon alpha and ribavirin alone or its combination diminished significantly. No significant independent risk factor of non-adherence to anti-HCV medications was detected. Delay in receiving new prescription was reported from patients as the most cause of non-adherence to both prescribed (peg) interferon alpha and ribavirin. Adherence to ribavirin was identified as an independent predictor of achieving ETR or SVR. These data could be used as a guide by health-care professionals and policy makers to develop optimal strategies for improving patient adherence to HCV treatment, enhancing virological as well as clinical outcome and allocating public resources properly.
